# Estimating Renal Function Following Lung Transplantation

**DOI:** 10.3390/jcm11061496

**Published:** 2022-03-09

**Authors:** Mads Hornum, Morten Baltzer Houlind, Esben Iversen, Esteban Porrini, Sergio Luis-Lima, Peter Oturai, Martin Iversen, Pia Bredahl, Jørn Carlsen, Christian Holdflood Møller, Mads Jønsson Andersen, Bo Feldt-Rasmussen, Michael Perch

**Affiliations:** 1 Department of Nephrology, Copenhagen University Hospital—Rigshospitalet, 2100 Copenhagen, Denmark; bo.feldt-rasmussen@regionh.dk; 2 Department of Clinical Medicine, University of Copenhagen, 2200 Copenhagen, Denmark; michael.perch@regionh.dk; 3 Department of Clinical Research, Copenhagen University Hospital—Amager and Hvidovre, 2650 Copenhagen, Denmark; morten.baltzer.houlind@regionh.dk (M.B.H.); esbeniversen1@gmail.com (E.I.); 4 The Capital Region Pharmacy, 2730 Herlev, Denmark; 5 Department of Drug Design and Pharmacology, University of Copenhagen, 2200 Copenhagen, Denmark; 6 Laboratory of Renal Function (LFR), Faculty of Medicine, University of La Laguna, 38200 La Laguna, Spain; esteban.l.porrini@gmail.com; 7 Faculty of Medicine, Instituto de Tecnologías Biomédicas (ITB), University of La Laguna, 38200 La Laguna, Spain; 8 IIS-Fundación Jiménez Diaz, Department of Medicine, School of Medicine, Universidad Autónoma de Madrid, 28049 Madrid, Spain; luis.lima.sergio@gmail.com; 9 Department of Clinical Physiology, Nuclear Medicine and PET, Copenhagen University Hospital—Rigshospitalet, 2100 Copenhagen, Denmark; peter.sandor.oturai@regionh.dk; 10 Department of Cardiology, Section for Lung Transplantation, Copenhagen University Hospital—Rigshospitalet, 2100 Copenhagen, Denmark; martin@iversen-net.dk; 11 Department of Cardiothoracic Anesthesiology, Copenhagen University Hospital—Rigshospitalet, 2100 Copenhagen, Denmark; pia.bredahl.jensen@regionh.dk; 12 Department of Cardiology, Copenhagen University Hospital—Rigshospitalet, 2100 Copenhagen, Denmark; joern.carlsen@regionh.dk (J.C.); madander@rm.dk (M.J.A.); 13 Department of Cardiothoracic Surgery, Copenhagen University Hospital—Rigshospitalet, 2100 Copenhagen, Denmark; christian.moeller.02@regionh.dk

**Keywords:** cystatin c, estimated glomerular filtration rate, immunosuppression, lung transplantation, measured glomerular filtration rate

## Abstract

Background: Patients undergoing lung transplantation (LTx) experience a rapid decline in glomerular filtration rate (GFR) in the acute postoperative period. However, no prospective longitudinal studies directly comparing the performance of equations for estimating GFR in this patient population currently exist. Methods: In total, 32 patients undergoing LTx met the study criteria. At pre-LTx and 1-, 3-, and 12-weeks post-LTx, GFR was determined by ^51^Cr-EDTA and by equations for estimating GFR based on plasma (P)-Creatinine, P-Cystatin C, or a combination of both. Results: Measured GFR declined from 98.0 mL/min/1.73 m^2^ at pre-LTx to 54.1 mL/min/1.73 m^2^ at 12-weeks post-LTx. Equations based on P-Creatinine underestimated GFR decline after LTx, whereas equations based on P-Cystatin C overestimated this decline. Overall, the 2021 CKD-EPI combination equation had the lowest bias and highest precision at both pre-LTx and post-LTx. Conclusions: Caution must be applied when interpreting renal function based on equations for estimating GFR in the acute postoperative period following LTx. Simplified methods for measuring GFR may allow for more widespread use of measured GFR in this vulnerable patient population.

## 1. Introduction

Graft and patient survival rates in lung transplantation (LTx) recipients have increased over the last few decades due to improved surgical procedures and early intensive treatment, but long-term outcomes remain worse than for other solid organ transplants [1,2]. Several factors influence short- and long-term outcomes after LTx. These include time after LTx, acute renal failure after LTx, use of heart and lung machines, use of perioperative ephedrine, older age, and impaired kidney function due to calcineurin inhibitor (CNI) toxicity or pre-LTx glomerular filtration rate (GFR) below 90 mL/min [3,4,5]. Early detection of renal impairment is important for implementing adaptive renal sparing strategies such as aggressive blood pressure control [6], CNI toxicity reduction [6,7,8], and dose adjustment for other renally excreted drugs [9,10]. However, only a few prospective studies have investigated reliable markers of renal function in this patient population [11,12] or other potential markers of acute kidney injury, such as soluble urokinase plasminogen activator receptor (suPAR) [13,14].

In clinical practice, renal function is commonly estimated with endogenous filtration markers such as plasma (P)-Creatinine, P-Cystatin C, or a combination of both. P-Creatinine is the most widely used, but it has several limitations, including reabsorption and secretion from renal tubular cells and extra-renal clearance. It is also affected by muscle mass and protein intake and is an insensitive marker for rapid GFR decline [15]. In general, GFR estimates based on P-Creatinine may be inaccurate and have large variability compared to measured GFR [16]. Creatinine-based estimates perform particularly poorly in patients with certain clinical conditions, such as diabetes, chronic kidney disease, renal transplantation, and lung transplantation [17,18].

In contrast, P-Cystatin C is largely independent of muscle mass and is commonly used to estimate GFR in pediatric patients with solid organ transplants [19]. During the past few years, equations for estimating GFR based on the combination of P-Creatinine and P-Cystatin C have been reported to be more accurate across diverse patient groups [20,21,22,23]. However, P-Cystatin C is affected by other factors such as obesity, inflammation, and use of immunosuppressive medications independent of the level of renal function [15,24,25,26,27]. To our knowledge, no studies have evaluated the acute changes in P-Cystatin C or compared the performance of different equations for estimating GFR based on P-Cystatin C before and after LTx. We measured GFR with ^51^Cr-EDTA clearance and determined the performance of equations for estimating GFR from the Chronic Kidney Disease Epidemiology Collaboration (CKD-EPI) with and without race [23,28,29], the Full Age Spectrum (FAS) [22,30], and the 2021 European Kidney Function Consortium (EKFC) [31]. We hypothesized that all GFR equations would have low accuracy, precision, and CKD class agreement compared to measured GFR shortly before and after LTx, but that equations combining P-Creatinine and P-Cystatin C would have the best performance.

## 2. Results

### 2.1. Patient Characteristics

In total, 39 patients were included in the original CARCATS study. For the current study, three patients were excluded due to no available blood samples at pre-LTx, and four patients were excluded due to lack of GFR measurement at pre-LTx (*n* = 1) or at more than one timepoint post-LTx (*n* = 3). Patient characteristics for the final study population (*n* = 32) are shown in Table 1. Mean age was 53.4 years, 59% of patients were male, and all were Caucasian. The most common etiology of lung disease was chronic obstructive pulmonary disease (COPD), which was present in 18 patients (56%). At pre-LTx, median P-Creatinine was 0.81 (IQR, 0.69–1.02) mg/dL and median P-Cystatin C was 0.90 (IQR, 0.77–0.98) mg/L. P-Thyroid stimulating hormone (TSH) was significantly lower at post-LTx compared to pre-LTx for both men (at 1 week) and women (at 1 week and 3 weeks) (Table S1). There were no significant changes in weight, body mass index (BMI), or body surface area (BSA) between pre-LTx and post-LTx.

### 2.2. Kidney Function over Time

P-Creatinine, P-Cystatin C, measured GFR, BMI, and BSA over time are shown in Figure S1. Kidney function according to P-Creatinine, P-Cystatin C, measured GFR, and estimated GFR over time is shown in Table 2. Median P-Creatinine and P-Cystatin C were 0.81 mg/dL and 0.90 mg/L at pre-LTx and increased to 0.98 mg/dL and 1.25 mg/L at 1-week post-LTx, 0.98 mg/dL and 1.37 mg/L at 3-weeks post-LTx, and 1.20 mg/dL and 1.42 mg/L at 12-weeks post-LTx, respectively. Median measured GFR according to ^51^Cr-EDTA clearance was 98.0 mL/min/1.73 m^2^ at pre-LTx and declined to 74.2, 62.3 and 54.1 mL/min/1.73 m^2^ at 1-, 3-, and 12-weeks post-LTx, respectively. Depending on choice of equation, median estimated GFR based on P-Creatinine was 82.9–96.6 mL/min/1.73 m^2^ at pre-LTx, 91.5–101.8 mL/min/1.73 m^2^ at 1-week post-LTx, 69.6–75.4 mL/min/1.73 m^2^ at 3-weeks post-LTx, and 58.6–61.7 mL/min/1.73 m^2^ at 12-weeks post-LTx. Median estimated GFR based on P-Cystatin C was 88.5–94.5 mL/min/1.73 m^2^ at pre-LTx, 56.6–61.1 mL/min/1.73 m^2^ at 1-week post-LTx, 55.5–60.0 mL/min/1.73 m^2^ at 3-weeks post-LTx, and 49.2–54.4 mL/min/1.73 m^2^ at 12-weeks post-LTx. Median estimated GFR based on the combination of P-Creatinine and P-Cystatin C was 83.4–94.9 mL/min/1.73 m^2^ at pre-LTx, 72.1–73.1 mL/min/1.73 m^2^ at 1-week post-LTx, 68.8–69.6 mL/min/1.73 m^2^ at 3-weeks post-LTx, and 51.8–57.5 mL/min/1.73 m^2^ at 12-weeks post-LTx. Sensitivity analysis excluding one patient with suspected AKI is shown in Table S2. A separate sensitivity analysis calculating GFR in BSA-adjusted units is shown in Table S3. Overall, median GFR was slightly higher in BSA-adjusted units because median BSA was slightly higher than 1.73 m^2^. 

### 2.3. Performance of Estimated GFR Equations 

Median bias, P30, P15, and CKD class agreement for each estimated GFR equation compared to measured GFR are shown in Table 3. At pre-LTx, all equations underestimated measured GFR with a median bias between 5.4 and 16.4 mL/min/1.73 m^2^. Across all timepoints post-LTx, equations based on P-Creatinine overestimated measured GFR with a median bias between −2.2 and −13.8 mL/min/1.73 m^2^, whereas equations based on P-Cystatin C underestimated measured GFR with a median bias between 1.2 and 15.7 mL/min/1.73 m^2^. Among all equations, bias was lowest for CKD-EPI_comb2021_ at pre-LTx (5.4 mL/min/1.73 m^2^), 1-week post-LTx (2.6 mL/min/1.73 m^2^), and 3-weeks post-LTx (0.4 mL/min/1.73 m^2^), and for FAS_comb_ at 12-weeks post-LTx (0.9 mL/min/1.73 m^2^).

At pre-LTx, equations based on P-Creatinine yielded a P30 between 84.4% and 93.8%, while equations based on P-Cystatin C yielded a P30 between 75.0% and 81.3%. At 1-week post-LTx, the EKFC_crea_ and CKD-EPI_comb2021_ equations yielded the highest P30 (83.3% and 80.0%, respectively), while the CKD-EPI_cys_ and FAS_cys_ equations yielded the lowest P30 (both 60.0%). At 3- and 12-weeks post-LTx, equations based on the combination of P-Creatinine and P-Cystatin C had the highest P30 (between 86.2% and 96.4%). 

Across all equations, P15 ranged from 43.9% to 56.2% at pre-LTx and from 37.5% to 64.3% at post-LTx. CKD class agreement ranged from 53.1% to 68.8% at pre-LTx, from 51.1% to 68.8% at 1-week post-LTx, from 24.1% to 69.0% at 3-weeks post-LTx, and from 50.0% to 60.7% at 12-weeks post-LTx. However, P15 accuracy and CKD class agreement post-LTx were never higher than 69.0% for any equation. 

Sensitivity analysis for the effect of randomization to felodipine vs. placebo did not reveal a significant association with bias for any estimated GFR equation (Table S4). Median bias for all equations was also largely unchanged in BSA-adjusted units (Table S5).

## 3. Discussion

This study is the first to directly compare the performance of the FAS, EKFC, and 2021 CKD-EPI equations both before and after surgery in patients undergoing lung transplantation. In the early postoperative period, equations based on P-Cystatin C alone were notably inaccurate compared to equations based on P-Creatinine alone, particularly at 1-week post-LTx. Equations based on P-Cystatin C appeared to overestimate the decline in GFR known to occur across LTx, whereas equations based on P-Creatinine appeared to underestimate this decline. Equations based on the combination of P-Creatinine and P-Cystatin C slightly underestimated the true decline at 1-week post-LTx but slightly overestimated the true decline at 12-weeks post-LTx. Overall, the CKD-EPI_comb2021_ equation had the lowest bias, highest accuracy, and highest CKD class agreement both pre- and post-LTx. Our results indicate that GFR estimates must be used and interpreted carefully when assessing GFR in patients undergoing LTx, particularly in the acute postoperative period.

### 3.1. Results in Context of Other Studies and Perspectives 

Earlier studies have demonstrated that including both P-Cystatin C and P-Creatinine in GFR equations improve GFR accuracy for both children and adults [23,28,32,33]. However, the findings have been inconsistent [34] and are often debated in the scientific literature. In pooled data from 20 pre-LTx and 77 post-LTx patients (median age 56 years, 47% male), Degen et al. found that the CKD-EPI_comb2012_ equation had the best performance with a P30 of 81%, P15 of 53%, and CKD class agreement of 72% compared to GFR measured with Tc-99 m diethylenetriaminepentaacetic acid (DTPA) [11]. A similar study by Florens et al. in 91 patients undergoing LTx (median age 47 years, 54% male) showed that the CKD-EPI_crea2009_ equation underestimated GFR compared to iohexol clearance, with a median bias of 18.7 mL/min/1.73 m^2^ and P30 of 64% at pre-LTx, and median bias of 5.0 mL/min/1.73 m^2^ and P30 of 85% at 1-year post-LTx [5]. These findings are very similar to our observations at pre-LTx, but we found that all equations overestimated measured GFR at 12-weeks post-LTx. However, the studies by Degen et al. and Florens et al. were not performed as consecutive prospective cohorts, and they did not evaluate the acute postoperative period [5,11]. Our results show poor performance of all nine estimated GFR equations at 1- and 3-weeks post-LTx, demonstrating that rapid decline in renal function after LTx is difficult to accurately detect with current endogenous filtration markers. 

Another important observation from our study is that equations based on P-Creatinine underestimated the rate of GFR decline following LTx, which is in concordance with a previous study by Broekroelofs et al. [12]. We have also previously described that postoperative administration of trimethoprim [3], which reduces tubular excretion of creatinine, could potentially lead to a reduction in the discrepancy between estimated and measured GFR at low levels [35]. LTx patients are at a high risk of change in muscle mass, which affects GFR estimates based on P-Creatinine. Therefore, it is not surprising that these equations underestimate GFR decline in the early postoperative period. Cystatin C is a low molecular weight protein that is easily filtered across the glomeruli [36] and reabsorbed and metabolized by tubular epithelial cells [28,37]. P-Cystatin C is independent of gender and muscle mass [15,38], but other factors such as inflammation immunosuppressants—common in LTx patients—are known to affect P-Cystatin C [15]. Patients using systemic steroids, for example, are known to have increased P-Cystatin C in a dose-dependent fashion [39]. The high level of baseline inflammation in this patient population may explain why equations based on P-Cystatin C underestimate GFR at all time points, and this should be taken into consideration in future studies. Given that patients typically receive high doses of systemic steroids after LTx, it is not surprising that GFR equations based on P-Cystatin C overestimate GFR decline. TSH levels are also known to affect P-Cystatin C [40,41]. A recent study in patients undergoing kidney transplantation found that elevated TSH levels were associated with a decline in GFR estimates based on P-Creatinine [42]. We found that TSH changed significantly between pre- and post-LTx (Table S1), which may also contribute to the inaccuracy of GFR estimates based on P-Cystatin C.

Change in body surface area (BSA) can also affect the long-term performance of both measured GFR and estimated GFR in BSA-unadjusted units. A recent study among patients with cancer in different stages of CKD found that the BSA-unadjusted CKD-EPI equation performed best overall [43]. We did not observe a significant change in weight, BMI, or BSA from pre-LTx to 12-weeks post-LTx (Table S1), so BSA adjustment is likely a minor issue in the early postoperative period. However, long-term changes in weight and BSA could introduce additional bias for BSA-unadjusted GFR. Therefore, calculating GFR in BSA-adjusted units may add more useful information in a clinical follow up, and we have provided these data in Table S3.

This study highlights the importance of carefully considering the choice of equations for estimating GFR in the early postoperative period following LTx. For example, our results indicate that equations based only on P-Cystatin C should be used with caution in the first 3 weeks following LTx. Equations combining P-Creatinine and P-Cystatin had the best performance in our cohort, but we did not assess the clinical relevance of this finding. However, it could be speculated that a more precise estimation of GFR with the combination equation in the postoperative period could lead to more efficient antiviral prophylaxis, since it is dose-dependent on GFR. For example, it has been documented for CMV that there is considerable risk of under- and overtreatment with antiviral medication in the early postoperative period [44]. We believe future studies should focus on identifying patients at high risk of rapid decline in GFR. Zacharias et al. developed a predictive model for CKD progression to renal failure based on six routine laboratory tests including P-Creatinine and P-Cystatin C. We speculate whether a similar model developed in patients undergoing LTx could be useful in clinical practice. One promising new biomarker is soluble urokinase plasminogen activator receptor (suPAR), which reflects systemic chronic inflammation and is strongly associated with GFR decline and acute kidney injury in various patient cohorts [45,46,47,48]. There may be potential for using suPAR to detect patients at high risk of rapid GFR decline following LTx, but this has not been investigated. Until accurate prediction models are developed to evaluate the risk of rapid GFR decline in patients undergoing LTx, we recommend assessing renal function based on measured GFR. We typically use ^51^Cr-EDTA, but other exogenous markers such as iohexol and inulin can also be considered. Previous studies [49] have shown excellent agreement between iohexol and inulin clearance for measuring GFR across different levels of renal function. Of course, the accuracy and precision of each method increases with the number of measurements collected, and patients with reduced GFR require a larger number of measurements. Accordingly, the procedure for measuring GFR requires up to 8 h in patients with GFR less than 30 mL/min. The procedure must also be performed with caution in patients with major edemas such as ascites, uncontrolled heart failure, or sepsis because iohexol can distribute into the extravascular space. In these cases, the procedure time can be even longer. Technical problems with intravenous injection can also be a barrier in patients with difficult venous accesses. Simplified methods for measuring iohexol clearance based on dried blood spot testing may reduce the procedure time and improve clinical feasibility of directly measuring GFR [50,51].

### 3.2. Strengths and Limitations

The strength of our study is the use of measured renal function using a gold standard method and inclusion of new equations for estimating GFR without race among a small but unique patient cohort undergoing LTx. This study also has several important limitations. First, the ^51^Cr EDTA method was developed in patients with varying degrees of renal function without edemas, in the non-fasting state. Therefore, this method relies on the assumption that ^51^Cr EDTA clearance from the total plasma volume can be calculated mathematically from a one-pool system based on the final slope of the ^51^Cr-EDTA plasma curve using a constant factor for correction [52]. The formula underestimates high values of clearance, for which a refinement of the formula has been derived [53]. Second, we did not collect urine samples or measure change in muscle mass due to LTx, which can affect the accuracy of equations based on P-Creatinine. We also could not determine the impact of steroid versus non-steroid treatment in this cohort.

## 4. Materials and Methods

### 4.1. Design and Study Cohort

This study is a secondary analysis of data collected for a randomized, double-blind, placebo-controlled clinical trial investigating acute renal complications after LTx at a national lung transplant center (trial name: CARCATS; clinicaltrials.gov identifier: NCT02744872; European Medicine Agency identifier: EudraCT 2008-004771-22). All patients provided informed written consent, and all secondary analyses were approved by the Danish Data Protection Agency (P-2020-657) and the Regional Ethical Review Board (H-20000528).

Details for the CARCATS cohort are described elsewhere [6]. In short, eligibility criteria for participant inclusion in the CARCATS study were the following: on the waiting list for single or double LTx, ≥18 years of age, no treatment with calcium channel blockers within the last 14 days, no known allergies to calcium channel blockers or placebo, and safe contraceptive use. Data were collected from January 2014 to July 2017 pre-LTx, and 1-, 3-, and 12-weeks post-LTx. Induction therapy with anti-thymocyte globulin (1.5 mg/kg daily) was given for the first 3 postoperative days. Azathioprine (1.5 mg/kg) was initiated at admission, and cyclosporine was initiated on the first postoperative day to obtain a trough level of 200–400 ng/mL for the first 3 months. Intravenous methylprednisolone was administered at anesthesia induction (1000 mg) and 4 times postoperatively (125 mg) at intervals of 8 h, and oral prednisolone (0.2 mg/kg) was initiated once daily starting on postoperative day 2. Patients were randomized 1:1 to receive felodipine or placebo before the start of the study. For the current study, patients were excluded if blood samples were not available at pre-LTx, or if GFR was not measured at pre-LTx or at more than one time point post-LTx.

### 4.2. P-Creatinine and P-Cystatin C Measurement

Blood samples were obtained and stored at −80 °C immediately prior to each GFR measurement (pre-LTx, 1-, 3-, and 12-weeks post-LTx). All biomarkers including P-Creatinine, P-Cystatin C, and P-thyroid stimulating hormone (TSH) were measured and calibrated at the Clinical Biochemical Department of Rigshospitalet on a Roche Cobas^®^ c 8000 e801. P-Creatinine was measured using the Creatinine Plus version 2 IDMS-traceable enzymatic assay (coefficient of variation 1.5%). P-Cystatin C was measured using the Roche Cystatin C Tina-quant generation 2 particle-enhanced immunonephelometric assay (coefficient of variation 2.2%). P-TSH was measured using a sandwich electrochemiluminescence-immunoassay (ECLIA) (coefficient of variation 4%).

### 4.3. Measured Glomerular Filtration Rate

Measured GFR was determined by ^51^Cr-EDTA clearance at baseline (pre-LTx) and 1-, 3- and 12-weeks post-LTx. The tracer was injected intravenously in an amount of 4 MBq, and two blood samples were collected from a cubital vein either 200 min after the injection if GFR was expected to be above 30 mL/min [54] or using the 4-point method if GFR was expected to be below 30 mL/min [52]. All GFR measurements were adjusted for body surface area (BSA) with the DuBois and DuBois formula [55].

### 4.4. Estimated Glomerular Filtration Rate

Estimated GFR was determined by the following equations: CKD-EPI based on P-Creatinine with race (CKD-EPI_crea2009_) or without race (CKD-EPI_crea2021_), P-Cystatin C (CKD-EPI_cys_), or the combination of P-Creatinine and P-Cystatin C with race (CKD-EPI_comb2012_) or without race (CKD-EPI_comb2021_) [23,28,29]; FAS based on P-Creatinine (FAS_crea_), P-Cystatin C (FAS_cys_), or the combination of P-Creatinine and P-Cystatin C (FAS_comb_) [22]; and EKFC based on P-Creatinine (EKFC_crea_) [31].

The CKD-EPI equations based on P-Creatinine were developed from 10 studies with 8254 participants and validated in 16 studies with 3896 participants. The equations were validated against GFR measured with iothalamate in the development cohort and iothalamate or other markers in the validation cohort, and linear regression was used to determine the influence of P-Creatinine, sex, race, and age [23,29]. The CKD-EPI equations based on P-Cystatin C or the combination of P-Creatinine and P-Cystatin were developed and validated in a similar manner [28]. The FAS equations were developed in 6870 children and adult patients with or without kidney disease and validated against measured GFR [30]. The EKFC equation was developed in 11,251 participants from 7 studies (development and internal validation) and validated in 8378 participants from 6 studies (external validation data set).

### 4.5. Outcomes and Statistical Analysis

Patient characteristics are presented in this paper with basic statistics: continuous variables are given as median with interquartile range, and discrete variables are given as numbers with percent of patients. Differences in selected patient characteristics between males and females were evaluated by Wilcoxon rank-sum test, and changes in selected patient characteristics over time were evaluated by Wilcoxon signed-rank test. Accuracy for each GFR equation was assessed by median bias (systematic error) compared to mGFR (mGFR-eGFR), percent of estimates within 30% (P30) or 15% (P15) of mGFR, and percent agreement in chronic kidney disease (CKD) classification compared to mGFR based on the following international staging guidelines: “normal or high GFR” (GFR > 90 mL/min/1.73 m^2^), “mildly decreased GFR” (GFR 60–89 mL/min/1.73 m^2^), “mildly to moderately decreased GFR” (GFR 45–59 mL/min/1.73 m^2^), “moderately to severely decreased GFR” (GFR 30–44 mL/min/1.73 m^2^), “severely decreased GFR” (GFR 15–29 mL/min/1.73 m^2^), or “kidney failure” (GFR < 15 mL/min/1.73 m^2^) [56]. Bootstrapping with 10,000 iterations was used to calculate confidence intervals for bias, P30, P15, and CKD class agreement. Sensitivity analysis based on a linear mixed-effects model was performed to determine whether randomization to felodipine or placebo had any influence on eGFR bias. Additional sensitivity analyses were performed to determine the impact of excluding patients with suspected AKI, or switching to GFR in BSA-adjusted units. For all statistical tests, *p* < 0.05 was considered statistically significant. All analyses were conducted in SAS Studio version 3.8 (SAS Institute, Cary, NC, USA).

## 5. Conclusions

In conclusion, we found that estimated GFR equations based on P-Creatinine overestimated measured GFR after LTx, whereas equations based on P-Cystatin C underestimated measured GFR after LTx. The CKD-EPI_comb2021_ equation had the lowest bias and highest P30 across LTx and may be the preferred equation to estimate GFR among patients undergoing LTx. However, P15 accuracy and CKD class agreement post-LTx were never higher than 69.0% for any equation. These findings indicate that caution must be applied when using estimated GFR to assess kidney function in patients undergoing LTx. In the early postoperative period, we suggest using a more reliable method for determining renal function, such as 99mTc-DTPA clearance or ^51^Cr-EDTA clearance.

## Figures and Tables

**Table 1:** Patient characteristics for all included patients (*n* = 32) pre-LTX.

Characteristic	
Female, *n* (%)	13 (40.6)
Age, median (IQR), years	53.4 (46.7–58.9)
Body mass index, median (IQR), kg/m^2^	20.9 (19.2–27.2)
P-Creatinine, median (IQR), mg/dL	0.81 (0.69–1.02)
P-Cystatin C, median (IQR), mg/L	0.90 (0.77–0.98)
Cr-EDTA clearance, ml/min/1.73 m*2*	98.0 (89.0–110.0)
Plasma cholesterol, median (IQR), mmol/L	5.22 (4.48–5.65)
Plasma TSH, median (IQR), IU/L	1.71 (0.99–2.16)

TSH, thyroid stimulating hormone.

**Table 2:** Median (IQR) filtration marker concentration and glomerular filtration rate (mL/min/1.73 m^2^) at each timepoint.

	Pre-LTx (*n* = 32)	1-Week Post-LTx (*n* = 30)	3-Week Post-LTx (*n* = 29)	12-Week Post-LTx (*n* = 28)
Filtration marker				
P-Creatinine, mg/dL	0.81 (0.69–1.02)	0.89 (0.71–1.15)	0.98 (0.83–1.49)	1.20 (1.07–1.57)
P-Cystatin C, mg/dL	0.90 (0.77–0.98)	1.25 (1/07–1.70)	1.37 (1.10–1.70)	1.42 (1.12–1.69)
Measured GFR	98.0 (89.0–110.0)	74.2 (55.3–96.2)	62.3 (48.2–80.5)	54.1 (48.3–72.3)
Creatinine-based eGFR				
CKD-EPI_crea2009	90.6 (74.9–105.5)	96.4 (54.9–104.3)	71.4 (58.7–95.4)	58.6 (46.5–78.0)
CKD-EPI_crea2021	96.6 (79.3–110.2)	101.8 (58.5–109.2)	75.4 (62.9–101.3)	61.7 (49.7–81.6)
FAS_crea	82.9 (73.3–108.0)	91.5 (54.4–104.3)	70.5 (58.3–91.8)	60.0 (47.6–77.2)
EKFC_crea	84.3 (75.0–103.3)	91.5 (53.1–99.1)	69.6 (57.5–92.0)	58.9 (45.8–75.8)
Cystatin C-based eGFR				
CKD-EPI_cys	94.2 (78.3–106.4)	56.6 (35.2–69.8)	55.0 (39.4–67.3)	49.2 (38.4–67.1)
FAS_cys	88.5 (68.9–96.3)	61.1 (38.6–72.0)	60.0 (45.3–66.6)	54.4 (44.6–65.0)
Creatinine-Cystatin C combined eGFR				
CKD-EPI_comb2012	89.4 (76.3–107.6)	72.1 (42.9–85.2)	68.8 (45.1–78.5)	51.8 (38.8–68.1)
CKD-EPI_comb2021	94.9 (78.7–112.0)	73.1 (44.3–86.8)	69.6 (46.4–77.9)	53.6 (39.9–71.5)
FAS_comb	83.4 (71.3–105.4)	72.4 (44.4–87.6)	69.5 (50.4–77.3)	57.5 (44.0–67.7)

CKD-EPI, Chronic Kidney Disease Epidemiology Collaboration; crea, Creatinine; comb, combination of Creatinine and Cystatin C; cys, Cystatin C, eGFR, estimated Glomerular Filtration Rate; EKFC, European Kidney Function Consortium; FAS, Full Age Spectrum; LTx, lung transplantation.

**Table 3:** (**A**) Median bias (95% CI) of eGFR equations compared with measured GFR at each timepoint, defined as mGFR–eGFR in normalized units (mL/min/1.73 m^2^). (**B**) P30 accuracy (95% CI) of eGFR equations compared with measured GFR at each timepoint, defined as percent of cases within 30% of mGFR. (**C**) P15 accuracy (95% CI) of eGFR equations compared with measured GFR at each timepoint, defined as percent of cases within 15% of mGFR. (**D**) CKD class agreement (95% CI) between eGFR equations and measured GFR at each timepoint, defined as percent of cases within the same CKD category.

(**A**)				
**Equation**	**Pre-LTx (*n* = 32)**	**1-Week Post-LTx** **(*n* = 30)**	**3-Week Post-LTx** **(*n* = 29)**	**12-Week Post-LTx** **(*n* = 28)**
Creatinine-based eGFR				
CKD-EPI_crea2009	11.8 (5.1 to 15.4)	−5.9 (−19.4 to –3.2)	−8.1 (−21.3 to −0.5)	−2.2 (−7.1 to 6.5)
CKD-EPI_crea2021	7.5 (0.3 to 10.9)	−10.5 (−22.9 to −6.4)	−13.8 (−25.0 to −3.6)	−5.0 (−11.6 to 2.3)
FAS_crea	6.1 (−1.3 to 15.9)	−9.2 (−17.3 to −1.2)	−4.2 (−23.2 to −1.6)	−3.6 (−9.2 to 3.7)
EKFC_crea	16.4 (8.3 to 20.3)	−2.7 (−12.3 to 0.2)	−4.7 (−16.7 to −0.2)	−2.6 (−6.3 to 6.6)
Cystatin C-based eGFR				
CKD-EPI_cys	9.4 (−0.8 to 15.4)	15.7 (8.6 to 25.1)	8.7 (4.5 to 14.0)	7.2 (1.1 to 13.7)
FAS_cys	12.3 (5.7 to 22.8)	12.5 (6.0 to 23.6)	7.2 (−0.7 to 14.4)	1.2 (−2.4 to 11.3)
Creatinine-Cystatin C combined eGFR				
CKD-EPI_comb2012	9.5 (3.4 to 18.0)	4.8 (−2.2 to 16.8)	3.1 (−2.6 to 5.9)	4.1 (0.1 to 8.1)
CKD-EPI_comb2021	5.4 (−1.0 to 14.0)	2.6 (−4.0 to 15.4)	0.4 (−4.4 to 2.1)	2.8 (−2.1 to 7.7)
FAS_comb	14.1 (3.4 to 19.6)	4.5 (−4.6 to 13.7)	2.3 (−4.3 to 6.3)	0.9 (−4.1 to 4.1)
(**B**)				
**Equation**	**Pre-LTx (*n* = 32)**	**1-Week Post-LTx** **(*n* = 30)**	**3-Week Post-LTx** **(*n* = 29)**	**12-Week Post-LTx** **(*n* = 28)**
Creatinine-based eGFR				
CKD-EPI_crea2009	84.4 (71.9 to 96.9)	80.0 (63.3 to 93.3)	72.4 (55.2 to 86.2)	78.6 (64.3 to 92.9)
CKD-EPI_crea2021	93.8 (84.4 to 100)	66.7 (50.0 to 83.3)	62.1 (44.8 to 79.3)	82.1 (67.9 to 96.4)
FAS_crea	87.5 (75.0 to 96.9)	76.7 (60.0 to 90.0)	65.5 (48.3 to 82.8)	82.1 (67.9 to 96.4)
EKFC_crea	90.6 (78.1 to 100)	83.3 (70.0 to 96.7)	79.3 (65.5 to 93.1)	82.1 (67.9 to 96.4)
Cystatin C-based eGFR				
CKD-EPI_cys	81.3 (65.6 to 93.8)	60.0 (43.3 to 76.7)	75.9 (58.6 to 89.7)	82.1 (67.9 to 96.4)
FAS_cys	75.0 (59.4 to 87.5)	60.0 (43.3 to 76.7)	89.7 (75.9 to 100)	85.7 (71.4 to 96.4)
Creatinine-Cystatin C combined eGFR				
CKD-EPI_comb2012	87.5 (75.0 to 96.9)	76.7 (60.0 to 90.0)	86.2 (72.4 to 96.6)	89.3 (75.0 to 100)
CKD-EPI_comb2021	96.9 (90.6 to 100)	80.0 (63.3 to 93.3)	86.2 (72.4 to 96.6)	92.9 (82.1 to 100)
FAS_comb	87.5 (75.0 to 96.9)	76.7 (60.0 to 90.0)	86.2 (72.4 to 96.6)	96.4 (89.3 to 100)
(**C**)				
**Equation**	**Pre-LTx (*n* = 32)**	**1-Week Post-LTx** **(*n* = 30)**	**3-Week Post-LTx** **(*n* = 29)**	**12-Week Post-LTx** **(*n* = 28)**
Creatinine-based eGFR				
CKD-EPI_crea2009	46.9 (31.2 to 65.6)	43.3 (26.7 to 60.0)	44.8 (27.6 to 62.1)	42.9 (25.0 to 60.7)
CKD-EPI_crea2021	50.0 (31.2 to 65.6)	46.7 (30.0 to 63.3)	37.9 (20.7 to 55.2)	42.9 (25.0 to 60.7)
FAS_crea	56.2 (37.5 to 71.9)	50.0 (33.3 to 66.7)	44.8 (27.6 to 62.1)	46.4 (28.6 to 64.3)
EKFC_crea	53.1 (34.4 to 68.8)	53.3 (36.7 to 70.0)	44.8 (27.6 to 62.1)	46.4 (28.6 to 64.3)
Cystatin C-based eGFR				
CKD-EPI_cys	56.2 (40.6 to 71.9)	30.0 (13.3 to 46.7)	55.2 (37.9 to 72.4)	50.0 (32.1 to 67.9)
FAS_cys	46.9 (28.1 to 65.6)	43.3 (26.7 to 60.0)	41.4 (24.1 to 58.6)	39.3 (21.4 to 57.1)
Creatinine-Cystatin C combined eGFR				
CKD-EPI_comb2012	53.1 (34.4 to 68.8)	46.7 (30.0 to 63.3)	51.7 (34.5 to 69.0)	53.6 (35.7 to 71.4)
CKD-EPI_ comb2021	53.1 (34.4 to 68.8)	43.3 (26.7 to 60.0)	48.3 (31.0 to 65.5)	53.6 (35.7 to 71.4)
FAS_comb	43.8 (28.1 to 62.5)	46.7 (30.0 to 63.3)	48.3 (31.0 to 65.5)	64.3 (46.4 to 82.1)
(**D**)				
**Equation**	**Pre-LTx (*n* = 32)**	**1-Week Post-LTx** **(*n* = 30)**	**3-Week Post-LTx** **(*n* = 29)**	**12-Week Post-LTx** **(*n* = 28)**
Creatinine-based eGFR				
CKD-EPI_crea2009	65.6 (50.0 to 81.2)	50.0 (33.3 to 66.7)	41.4 (24.1 to 58.6)	57.1 (39.3 to 75.0)
CKD-EPI_crea2021	59.4 (43.8 to 75.0)	53.3 (36.7 to 70.0)	24.1 (10.3 to 41.4)	50.0 (32.1 to 67.9)
FAS_crea	68.8 (53.1 to 84.4)	56.7 (40.0 to 73.3)	51.7 (34.5 to 69.0)	53.6 (35.7 to 71.4)
EKFC_crea	65.6 (50.0 to 81.2)	50.0 (33.3 to 66.7)	51.7 (34.5 to 69.0)	53.6 (35.7 to 71.4)
Cystatin C-based eGFR				
CKD-EPI_cys	65.6 (50.0 to 81.2)	36.7 (20.0 to 53.3)	51.7 (34.5 to 69.0)	53.6 (35.7 to 71.4)
FAS_cys	53.1 (37.5 to 68.8)	40.0 (23.3 to 56.7)	62.1 (44.8 to 79.3)	53.6 (35.7 to 71.4)
Creatinine–Cystatin C combined eGFR				
CKD-EPI_comb2012	62.5 (46.9 to 78.1)	50.0 (33.3 to 66.7)	69.0 (51.7 to 86.2)	60.7 (42.9 to 78.6)
CKD-EPI_ comb2021	68.8 (53.1 to 84.4)	50.0 (33.3 to 66.7)	69.0 (51.7 to 86.2)	57.1 (39.3 to 75.0)
FAS_comb	62.5 (46.9 to 78.1)	36.7 (20.0 to 53.3)	65.5 (48.3 to 82.8)	57.1 (39.3 to 75.0)

CKD-EPI, Chronic Kidney Disease Epidemiology Collaboration; crea, Creatinine; comb, combination of Creatinine and Cystatin C; cys, Cystatin C, eGFR, estimated Glomerular Filtration Rate; EKFC, European Kidney Function Consortium; FAS, Full Age Spectrum; LTx, lung transplantation.

## Data Availability

Data are available on request due to restrictions. The data presented in this study are not publicly available due to Danish legislation. Request to access the dataset will require an individual inquiry to the Danish Data Protection agency for approval.

## Supplementary Materials

The following supporting information can be downloaded at: https://www.mdpi.com/article/10.3390/jcm11061496/s1. Figure S1. Spaghetti plots of P-Creatinine (A), P-Cystatin C (B), measured GFR (C), body mass index (D), and body surface area (E) over time. Bold lines represent median values at each timepoint. Dotted line shown for body surface area of 1.73 m^2^ as reference. Table S1. Median (IQR) values for selected patient characteristics at each timepoint. Table S2. Median (IQR) glomerular filtration rate (mL/min/1.73 m^2^) over time, excluding one patient with suspected acute kidney injury. Table S3. Median (IQR) glomerular filtration rate in BSA-adjusted units (mL/min) at each timepoint. Table S4. Sensitivity analysis for association between eGFR equation bias and randomization to felodipine vs. placebo. Table S5. Median bias (95% CI) of eGFR equations compared with measured GFR at each timepoint, defined as mGFR–eGFR in BSA-adjusted units (mL/min).
