# Supplementary material for: Estimating Renal Function Following Lung Transplantation
Source: J Clin Med. 2022 Mar 9;11(6):1496. doi: 10.3390/jcm11061496 (PMC8956010; doi:10.3390/jcm11061496)
Supplement: Supplementary file 1 [file jcm-11-01496-s001.zip › jcm-1595801-supplementary.pdf]

Journal of
Clinical Medicine

Supplementary Material

(A)

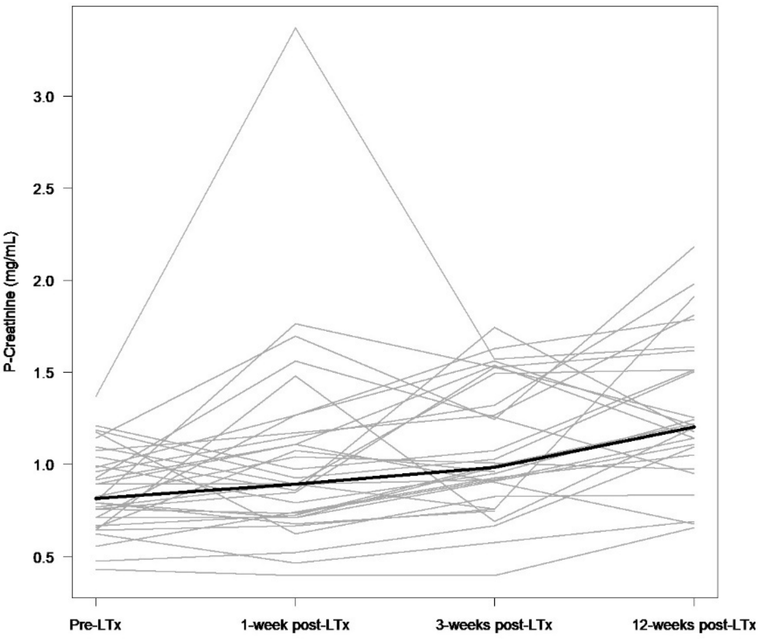

(B)

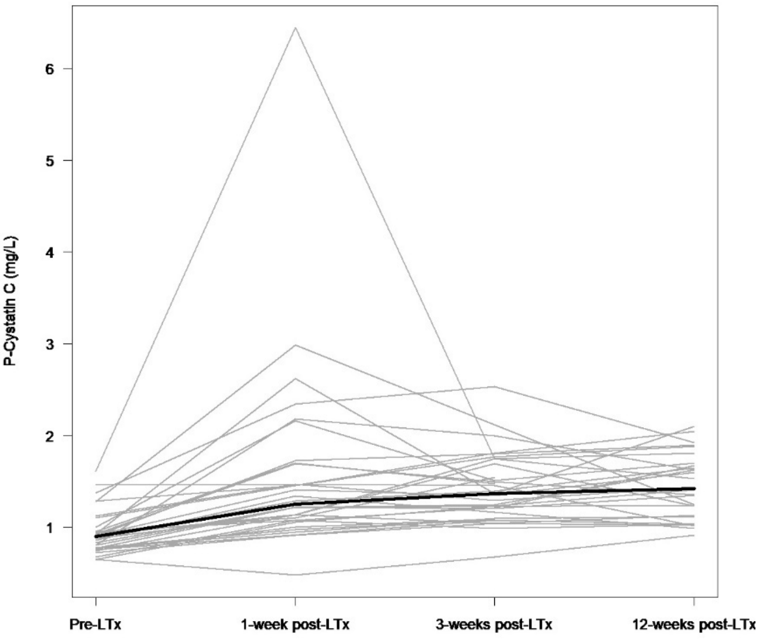

(C)

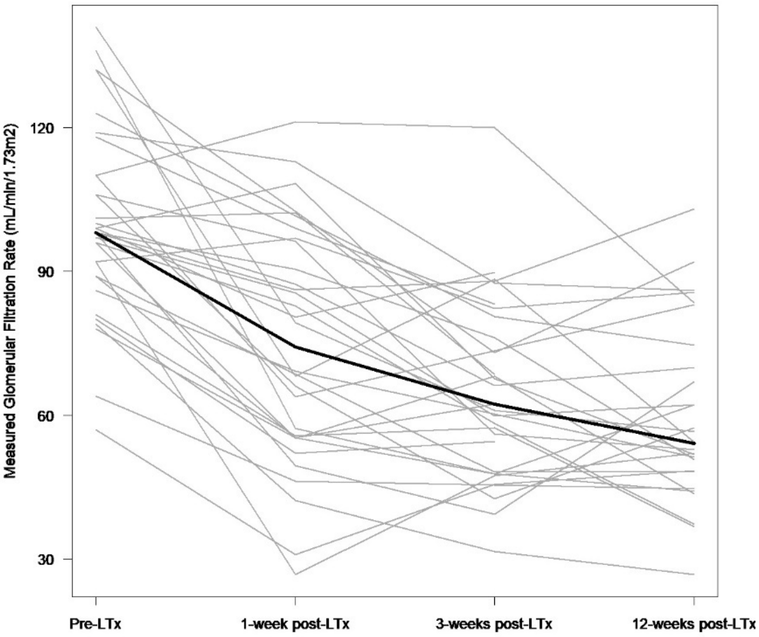

Measured Glomerular Filtration Rate (mL/min/1.73m2)
120
90
60
30
Pre-LTx
1-week post-LTx
3-weeks post-LTx
12-weeks post-LTx

(D)

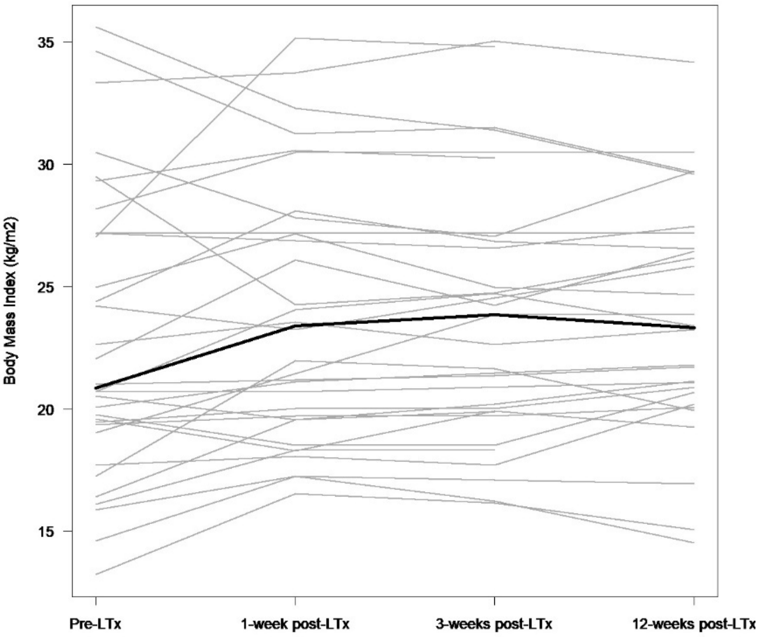

Body Mass Index (kg/m2)
35
30
25
20
15
Pre-LTx
1-week post-LTx
3-weeks post-LTx
12-weeks post-LTx

(E)

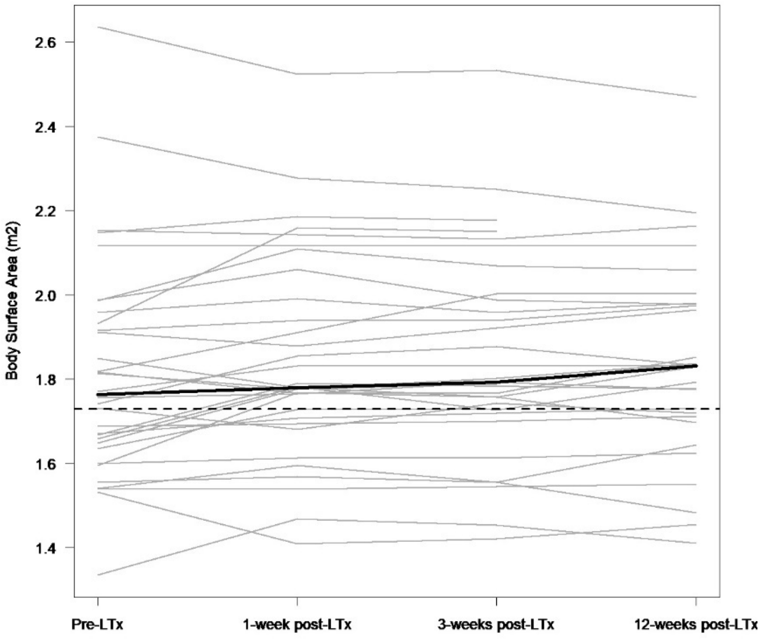

Figure S1. Spaghetti plots of P-Creatinine (A), P-Cystatin C (B), measured GFR (C), body mass index (D), and body surface area (E) over time. Bold lines represent median values at each timepoint. Dotted line shown for body surface area of 1.73 m^2 as reference

Table S1. Median (IQR) values for selected patient characteristics at each timepoint.

Variable	**Pre-LTx**	**1-week post-LTx**	**3-week post-LTx**	**12-week post-LTx**
Weight, kg	64 (54–80)	71 (58–87)	70 (61–82)	71 (59–80)
Body mass index, kg/m^2	20.9 (19.2–27.2)	23.4 (19.6–27.8)	23.9 (19.9–27.1)	23.3 (20.4–26.9)
Body surface area, m^2	1.76 (1.64–1.94)	1.78 (1.71–2.06)	1.79 (1.73–2.00)	1.83 (1.70–1.98)
P-TSH, IU/L	F: 1.78 (1.48–3.10)<br>M: 1.55 (0.96–1.83)	F: 0.35 (0.15–1.02)*<br>M: 0.65 (0.29–1.24)*	F: 1.18 (0.42–1.50)*<br>M: 1.15 (0.89–1.71)	F: 1.45 (0.69–1.95)<br>M: 1.59 (0.86–2.23)

IQR: inter quartil range, F, female; M, male; TSH, thyroid stimulating hormone; LTx: lung transplantation, *significant ($p<0.05$) difference from pre-LTx.

Table S2. Median (IQR) glomerular filtration rate (mL/min/1.73m^2) over time, excluding one patient with suspected acute kidney injury

	**Pre-LTx** (n = 31)	**1-week post-LTx** (n = 29)	**3-week post-LTx** (n = 28)	**12-week post-LTx** (n = 27)
Measured GFR	98.0 (89.0–110.0)	79.2 (55.3–96.2)	64.3 (51.4–81.4)	54.3 (48.2–74.6)
Creatinine-based eGFR				
CKD-EPI_crea2009	93.7 (74.9–106.9)	96.4 (57.0–104.3)	77.7 (59.5–95.7)	60.3 (46.7–82.5)
CKD-EPI_crea2021	99.4 (79.5–111.2)	101.8 (60.2–109.2)	82.3 (63.0–101.5)	63.5 (49.9–85.5)
FAS_crea	85.5 (74.0–108.0)	91.7 (62.7–104.3)	73.3 (58.3–92.0)	62.1 (48.1–77.3)
EKFC_crea	85.9 (75.2–103.4)	91.5 (59.1–99.1)	74.4 (57.6–92.4)	59.5 (46.2–76.7)
Cystatin C-based eGFR				
CKD-EPI_cys	94.8 (80.8–108.1)	57.3 (43.0–69.8)	56.0 (40.9–67.6)	49.9 (38.6–68.3)
FAS_cys	89.3 (68.9–96.3)	61.1 (45.2–72.0)	60.4 (44.9–66.7)	54.9 (42.7–65.2)
Creatinine-Cystatin C combined eGFR				
CKD-EPI_comb2012	89.8 (77.8–107.9)	73.3 (46.9–85.2)	68.9 (47.9–79.9)	52.9 (38.9–68.4)
CKD-EPI_comb2021	95.0 (81.6–112.0)	74.8 (49.0–86.8)	69.8 (49.4–81.2)	54.5 (40.0–71.9)
FAS_comb	84.4 (71.9–107.8)	72.5 (48.0–87.6)	70.2 (50.7–77.5)	58.4 (43.2–68.1)

Table S3. Median (IQR) glomerular filtration rate in BSA-adjusted units (mL/min) at each timepoint

	**Pre-LTx** (n = 31)	**1-week post-LTx** (n = 29)	**3-week post-LTx** (n = 28)	**12-week post-LTx** (n = 27)
Measured GFR	98.3 (85.0–125.5)	79.6 (56.5–102.7)	70.8 (55.8–83.9)	58.5 (50.0–77.0)
Creatinine-based eGFR				
CKD-EPI_crea2009	95.2 (77.1–115.4)	102.9 (57.5–117.0)	83.3 (63.1–103.0)	60.8 (47.9–87.0)
CKD-EPI_crea2021	99.5 (82.6–117.5)	108.3 (61.2–122.8)	88.4 (67.6–110.5)	64.7 (51.3–91.9)
FAS_crea	94.8 (74.4–119.9)	102.9 (61.8–121.0)	85.9 (66.4–98.2)	65.1 (50.3–80.4)
EKFC_crea	91.9 (75.6–105.5)	98.8 (59.3–109.4)	83.8 (64.0–96.9)	62.1 (47.3–83.1)
Cystatin C-based eGFR				
CKD-EPI_cys	94.3 (79.8–106.2)	59.8 (40.3–76.2)	57.6 (45.2–66.7)	51.6 (38.1–69.6)
FAS_cys	91.2 (76.3–99.8)	64.5 (46.2–75.7)	63.4 (52.1–69.4)	58.2 (48.2–67.8)
Creatinine-Cystatin C combined eGFR				
CKD-EPI_comb2012	96.2 (81.1–107.7)	77.2 (48.0–94.4)	68.2 (52.2–80.0)	55.0 (43.5–74.4)
CKD-EPI_comb2021	100.3 (86.0–110.4)	78.5 (50.1–96.8)	70.0 (53.7–80.8)	56.8 (44.0–77.5)
FAS_comb	91.9 (76.6–108.1)	79.7 (52.9–91.4)	73.6 (54.2–83.9)	62.3 (50.7–71.1)

Table S4. Sensitivity analysis for association between eGFR equation bias and randomization to felodipine vs placebo

Equation	Estimate (95% CI)	P-value
Creatinine-based eGFR		
CKD-EPI_crea2009	4.27 (–6.5 to 15.0)	0.43
CKD-EPI_crea2021	3.81 (–7.0 to 14.6)	0.49
FAS_crea	6.36 (–5.1 to 17.8)	0.27
EKFC_crea	3.15 (–7.1 to 13.4)	0.54
Cystatin C-based eGFR		
CKD-EPI_cys	6.55 (–4.4 to 17.5)	0.24
FAS_cys	6.83 (–3.6 to 17.3)	0.20
Creatinine-Cystatin C combined eGFR		
CKD-EPI_comb2012	6.23 (–4.4 to 16.9)	0.25
CKD-EPI_comb2021	6.26 (–4.5 to 17.0)	0.25
FAS_comb	6.58 (–3.8 to 16.9)	0.21

Performed using a linear mixed-effect model with form mGFR = eGFR + β_1(Randomization) + β_2(Randomization × eGFR). "Estimate" and "P-value" are shown for β_2.

Table S5. Median bias (95% CI) of eGFR equations compared with measured GFR at each timepoint, defined as mGFR–eGFR in BSA-adjusted units (mL/min)

Equation	**Pre-LTx** (n = 32)	**1-week post-LTx** (n = 30)	**3-week post-LTx** (n = 29)	**12-week post-LTx** (n = 28)
Creatinine-based eGFR				
CKD-EPI_crea2009	11.7 (5.3 to 17.1)	–6.6 (–18.3 to –3.4)	–9.9 (–21.3 to –0.8)	–2.3 (–7.7 to 7.1)
CKD-EPI_crea2021	7.8 (0.4 to 12.0)	–12.1 (–21.8 to –7.6)	–14.4 (–24.9 to –4.4)	–5.4 (–12.7 to 2.4)
FAS_crea	6.7 (–1.4 to 16.2)	–9.7 (–17.1 to –1.3)	–5.4 (–23.6 to –1.9)	–3.6 (–9.8 to 4.0)
EKFC_crea	15.8 (8.4 to 20.4)	–2.5 (–12.4 to 0.3)	–5.3 (–18.9 to –0.2)	–2.8 (–6.8 to 7.1)
Cystatin C-based eGFR				
CKD-EPI_cys	10.6 (–0.6 to 19.0)	16.9 (9.1 to 29.1)	9.8 (5.1 to 15.0)	7.7 (1.2 to 14.8)
FAS_cys	13.7 (5.2 to 26.9)	13.6 (5.7 to 27.1)	7.2 (–1.0 to 15.2)	1.2 (–2.8 to 10.0)
Creatinine-Cystatin C combined eGFR				
CKD-EPI_comb2012	10.3 (3.3 to 19.1)	5.6 (–2.3 to 18.3)	3.2 (–2.9 to 7.2)	4.2 (0.2 to 9.3)
CKD-EPI_comb2021	6.0 (–0.9 to 15.6)	3.1 (–4.1 to 16.8)	0.6 (–4.5 to 2.6)	2.8 (–1.9 to 8.3)
FAS_comb	14.0 (3.7 to 21.3)	5.5 (–4.2 to 15.1)	2.3 (–4.3 to 8.0)	1.0 (–4.1 to 4.8)

CKD-EPI , Chronic Kidney Disease Epidemiology Collaboration; crea, creatinine; comb, combination of creatinine and cystatin c; cys, cystatin C, eGFR, estimated Glomerular Filtration Rate; EKFC, European Kidney Function Consortium; FAS, Full Age Spectrum; LTx, Lung transplantation.
